# Objective quantitative digital evaluation of crown preparation using intraoral optical scanner: Novel preclinical teaching method

**DOI:** 10.4317/jced.62125

**Published:** 2024-10-01

**Authors:** Bahram Ranjkesh, Golnoush Bahrami

**Affiliations:** 1 Section for Prosthetic Dentistry, Department of Dentistry and Oral Health, Aarhus University, Aarhus, Denmark

## Abstract

**Background:**

Adequate preclinical training in dentistry is crucial for students before engaging in patient treatment. Constructive feedback from the instructors plays a pivotal role in guiding the students to master skills, boost confidence, and above all ensure patient safety. This manuscript introduces a new method employing intraoral scanners as digital tools to provide objective and quantitative feedback for crown preparation evaluation in preclinical prosthodontics.

**Material and Methods:**

Initial reference scan before preparation on typodont and preparation scan after crown preparation was obtained. Utilizing the patient monitoring feature in Trios 3 scanner (3Shape, Copenhagen, Denmark) superimposition of two scans was performed.

**Results:**

The method enabled a comprehensive quantitative preparation assessment compared to the tooth pre-preparation. This includes tooth reduction in any axis, abutment height measurement, evaluation of the status of adjacent teeth after preparation, and proposed capability for estimating the convergence angle.

**Conclusions:**

The method enabled a subjective quantitative evaluation of crown preparation in phantom model. This method requires no additional hardware or software beyond the basic functions embedded in the intraoral scanner. Implementation of this function not only facilitates feedback and self-evaluation for students during preclinical teaching but also enhances their proficiency in using intraoral scanners in clinical practice in perspective.

** Key words:**Dental education, prosthodontics, intraoral scanner, feedback, self-assessment.

## Introduction

Dental education involves teaching and shaping future dental professionals, and educating them to prevent, diagnose, and treat oral diseases in the community. The educational framework typically integrates theoretical and clinical components, with the overarching objective of cultivating dentists capable of delivering evidence-based and safe treatments in society. This recognition highlights the need for a comprehensive understanding of the challenges associated with clinical training to enhance the overall efficacy of dental education programs (1,2). Preclinical rehearsal instruction is fundamental for developing the dexterity and technical skills of dental students. This skill development contributes to increased self-confidence, positively impacting their efficacy and patient safety in clinical treatment, academic performance, and stress management. Recognizing the pivotal role of preclinical rehearsal is essential for optimizing educational outcomes (3). Assessments are usually the main focus for students and the driving force for them to engage in the learning process. Therefore, it is recommended that “assessment processes should be rigorous, appropriate and reliable as a gateway for dental graduates to become qualified to practice independently” (4).

In prosthodontics, the preparation for indirect restoration, typically a full coverage crown is one of the fundaments in every teaching curriculum. The preparation necessitates the sufficient reduction of tooth structure to ensure the crown material exhibits optimal thickness, strength, and appropriate contour, while still respecting the biological limitations. Striking a balance between achieving these essential parameters and conserving tooth structure poses a challenge, where the retention and resilience form in preparation should follow traditional disciplines (5). In numerous dental schools, students routinely prepare artificial acrylic teeth in a preclinical setup, where students receive feedback during practical classes followed by summative feedback. Ensuring objectivity and consistency in the assessment and feedback process is imperative and despite assessor calibration efforts, conventional assessments relying on visual inspection have demonstrated inherent subjectivity and inconsistency (6-8). Addressing these challenges is crucial for enhancing the reliability and validity of practical examinations in dental education. Various educational digital tools and software such as E4D software (8), KaVo PREP assistant system (9), and prepCheck system (10) were introduced to overcome the problem; however, depending on the type of the system, each system requires special software or additional hardware, which is not in clinical practice, and are costly to purchase. Digital dentistry is becoming commonplace and has revolutionized the everyday dental practice. Intraoral optical scanners (IOS) have been fundamental in this digital transition by facilitating the direct acquisition of intraoral data (11). This transition emphasis the need of updating the curriculum in dental education. The newer generation of IOS has featured new technology to superimpose two digital scans to monitor a patient’s dentitional changes over time such as tooth wear (12,13). In this article, we introduce a new method for application of this feature as a digital tool in evaluating crown preparation in a preclinical prosthodontic.

## Material and Methods

This technique can be used for the preparation procedure of artificial teeth for full or partial coverage crowns or fixed partial dentures to control. In this method, we have used Trios 3 (3Shape, Copenhagen, Denmark) intraoral optic scanner with patient monitoring interface that is included in the Trios 3 standard subscription. The typodont with acrylic artificial teeth with a full crown preparation of tooth 47 is demonstrated in this method.

Initial reference scan 

**Figure 1:**
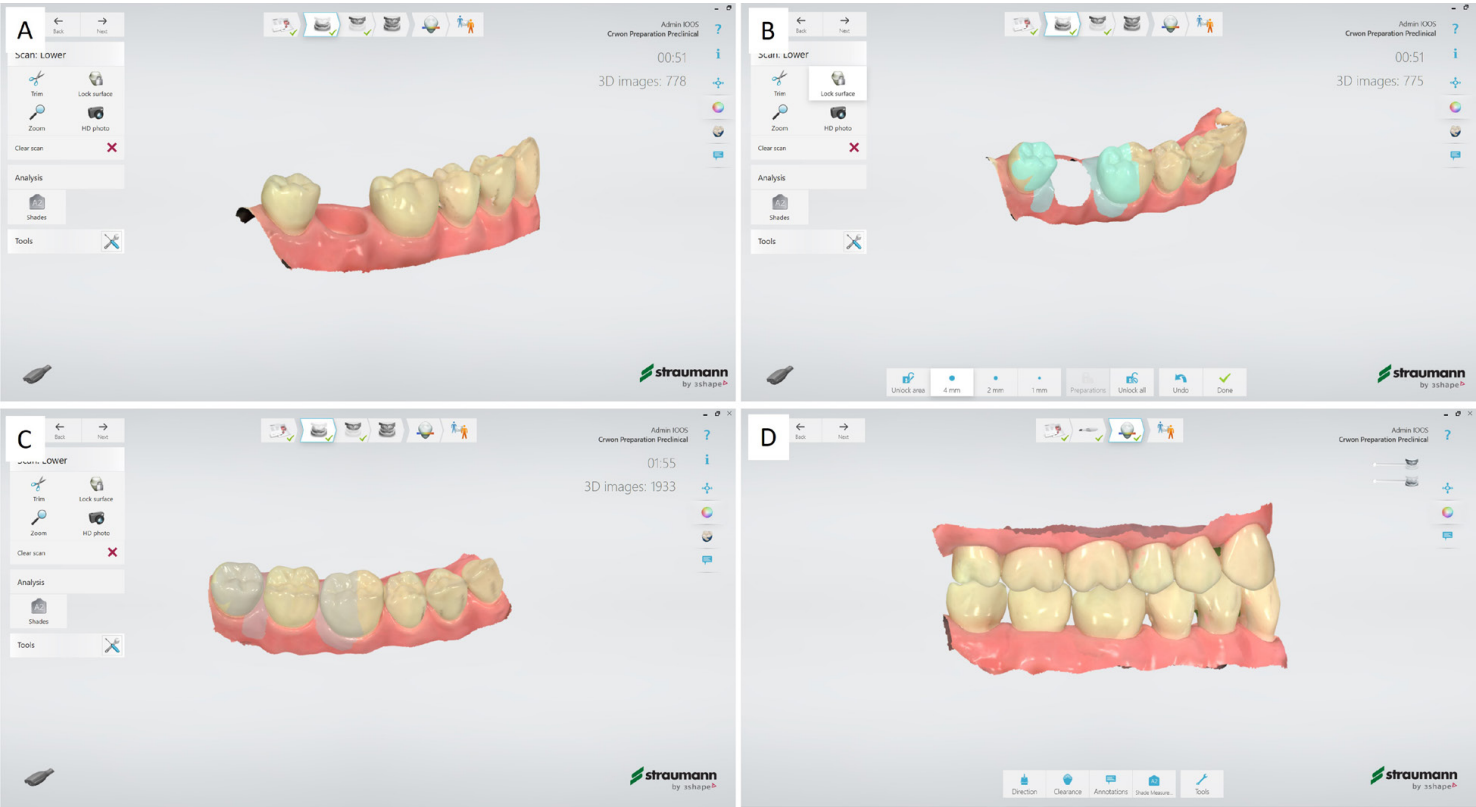
Initial reference scan. A. Scanning of typodont without tooth 47 in place to obtain adjacent teeth proximal surfaces. B. Area cut-off and surface lock tooth. C. Scanning of the typodont with tooth 47 places. D. post-processing of the lower-, upper-jaw and bite scans.

1. Place and fix all teeth in the typodont except tooth 47.

2. Run the Unit program in the Trios 3 scanner and create the order for initial scan in Scan only mode.

3. Scan the segment without tooth 47 in place to thoroughly adjacent teeth (Fig. 1A).

4. Cut-off the area from the scan area of tooth 47 and surface-lock the adjacent teeth (Fig. 1B).

5. Place the tooth 47 in typodont and scan the segment (Fig. 1C).

6. Scan the upper jaw and bite, and postprocess the scans (Fig. 1D).

Preparation scan

**Figure 2:**
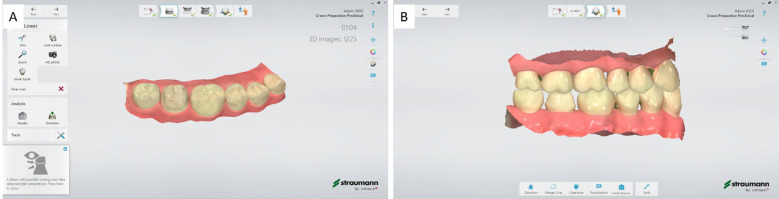
Preparation scan. A. Scanning of the typodont after crown preparation of tooth 47 and B. post-processing of the lower-, upper-jaw and bite scans.

7. Create a new order according to the restoration type.

8. Scan the segment after full coverage crown preparation of tooth 47 (Fig. 2A).

9. Scan the upper jaw and bite, and postprocess the scans (Fig. 2B).

Superimposition of initial and preparation scans

**Figure 3:**
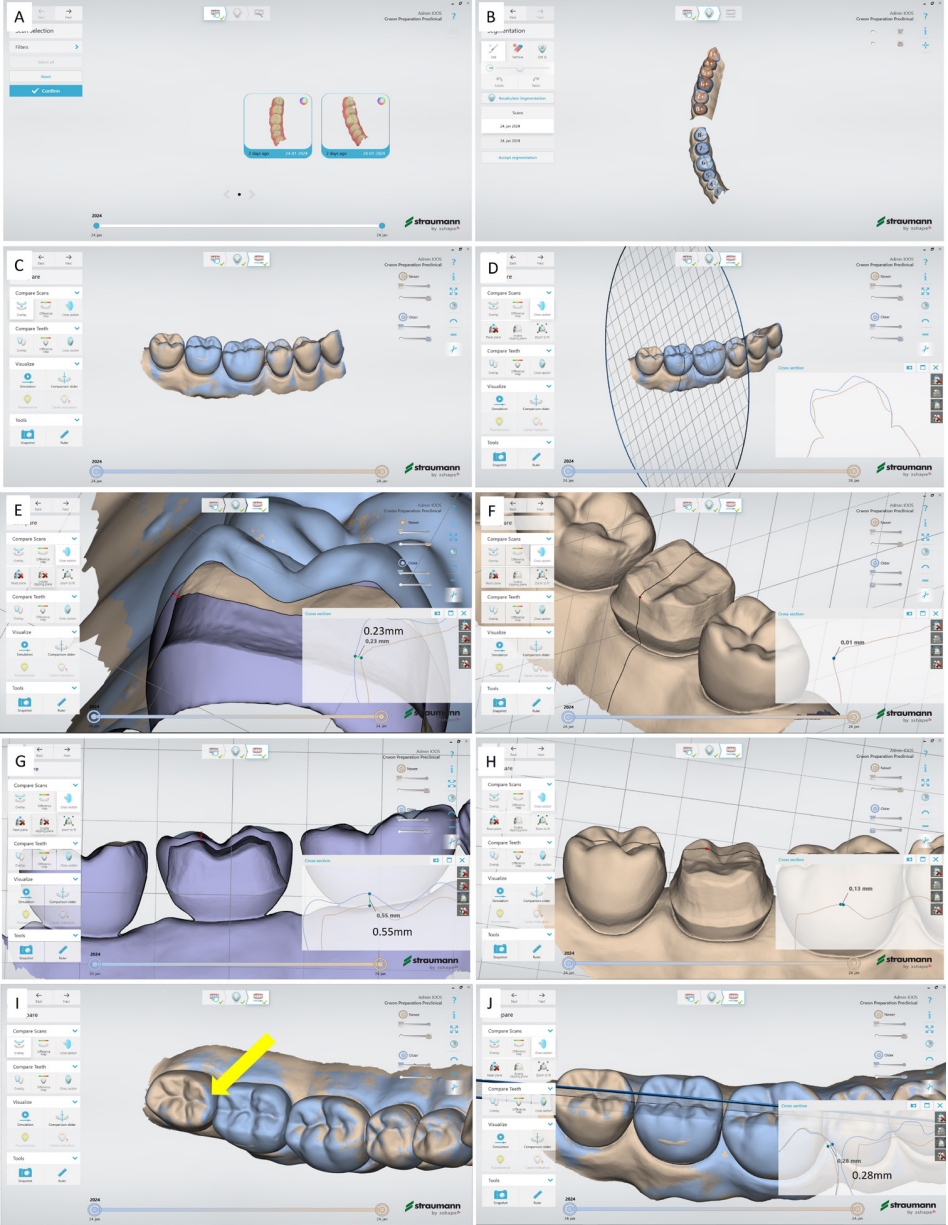
Superimposition of initial and preparation scan using Patient monitoring feature in Trios 3 IOS. A. Selecting the initial and preparation scans. B. Confirmation of segmentation. C. Superimposition of initial (blue color) and preparation (brown color) scans. Inspection and quantitative evaluation of the preparation in comparison to initial reference scan in buccolingual (E and F) and mesiodistal (G and H) directions. I and J. Intentional bur damage (280 µm) to mesial surface of tooth 48 (yellow arrow) during preparation.

**Figure 4:**
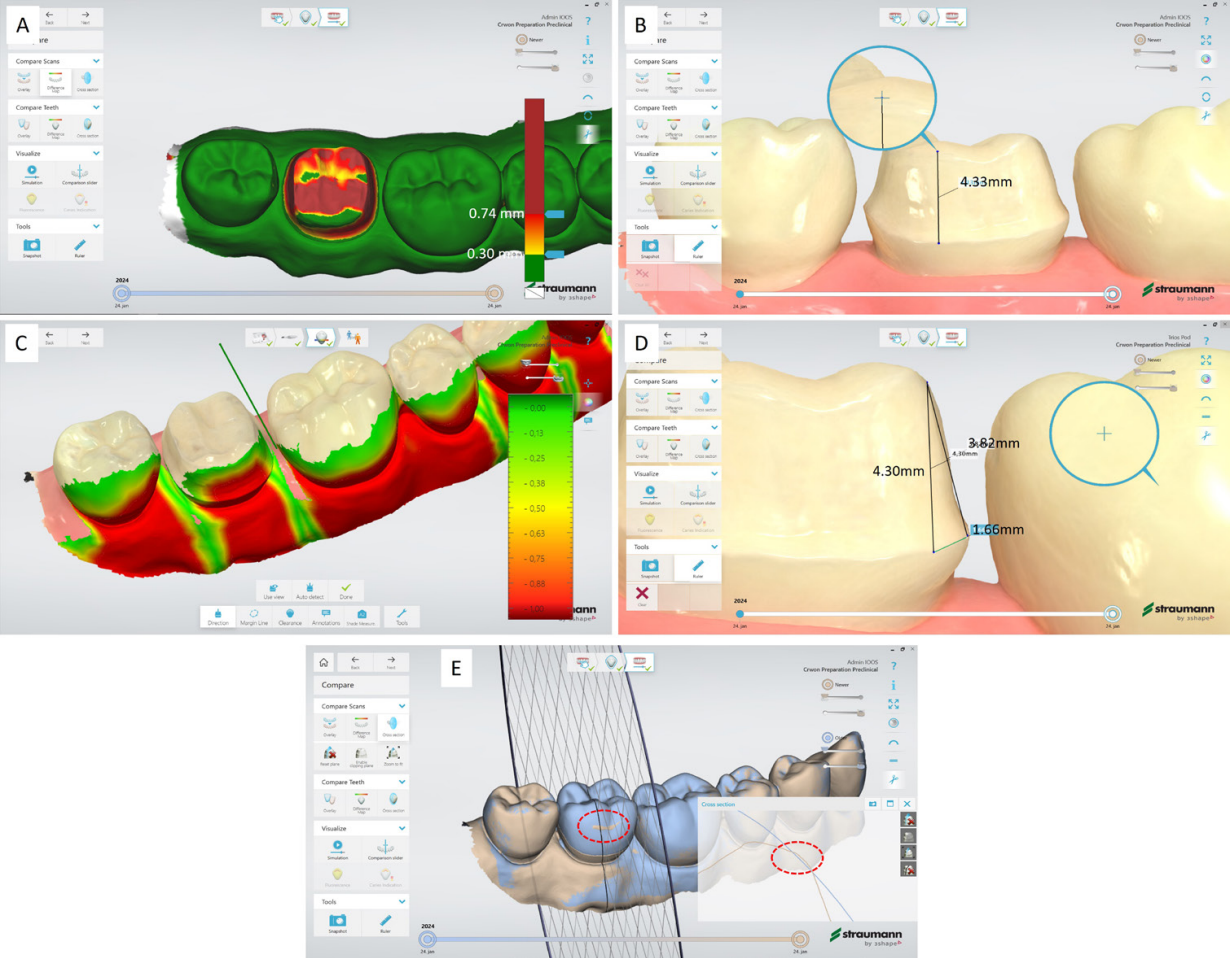
Color mapping illustrates the general quantitative reduction in preparation compared to the initial scan. B. Measurement of the cervico-occlusal height of the prepared tooth. C. Control of path of insertion and undercut evaluation by aid of the color scale. D. Proposed model to estimate the convergence angle between tooth axis and axial tapered surface by calculating the length of three lines of tooth axis, axial surface, and connecting line between these two. E. Improper superimposition of initial and preparation scan in the facial surface of tooth 47 marked in dashed circle that may indicate the need for software improvement.

10. Create new order using Patient Monitoring feature in Trios 3 scanner.

11. Select and confirm the initial reference and preparation scans (Fig. 3A).

12. Control, correct (if it is necessary), and confirm the segmentation (Fig. 3B)

13. Superimposition of initial (blue color) and preparation (brown color) scans after deselecting the upper jaw scans (Fig. 3C).

14. Select the ‘Cross section’ function under ‘Compare Scans’ menu and draw the desired section line to evaluate. The cross section and difference between two scans are observable in ‘Cross section’ window (Fig. 3D).

15. The reduced tooth substance is measurable in millimeters by clicking on two scans on ‘Cross section’ window. The corresponding point is observable by enabling the ‘Disable clipping plane’ function (Fig. 3E).

16. Deactivate the ‘Disable clipping plane’ to precisely see the under prepared area that requires further occlusal reduction (Fig. 3F).

17. By cutting plan in mesiodistal direction the occlusal reduction in corresponding direction is measurable (Fig. 3G,H). Note the sharp distal axio-occlusal line angle in the preparation that requires further smoother and rounded line angle (Fig. 3G).

18. Damage to the adjacent tooth 48 during preparation of tooth 47 is measurable by the same function (Fig. 3I,J).

19. Enabling the ‘Difference map’ allows the general quantitative reduction in preparation compared to the unprepared tooth. Note the bur damage to tooth 48 is not observable in color map since the threshold for difference illustration is 300 µm (Fig. 4A)

20. The gingiva-occlusal height is measurable to evaluate retentive form of preparation as the minimum abutment height (Fig. 4B).

## Discussion

In this manuscript, we have introduced the e new method to utilize the patient monitoring function in IOS for the quantitative assessment of crown preparation as a pedagogical tool in preclinical teaching.

The method is using the unprepared intact artificial tooth as the reference, which is then superimposed and compared to prepared tooth for full crown after taking the digital impression using IOS. The minimum required tooth reduction occlusally, cervically, and axially depends on the type of restoration. Selecting the desired cutting plan on ISO provides the possibility of evaluation of sufficient tooth reduction according to the restoration type in any axis. In contrast to traditional putty-index for preparation check where the method is destructive and number of cuts is very limited, there is no cut-plan limitation in the non-destructive digital model. Combining this tool with the restoration path of insertion (Fig. 4C) and assessing occlusal clearance enables a nearly comprehensive quantitative evaluation of tooth preparation using the standard functions of an intraoral optical scanner (IOS). The occlusal clearance tool in IOS may be highly valuable for clinically evaluating occlusal reduction in consideration of the patient’s habitual occlusion. Achieving a sTable occlusion in a typodont during preclinical teaching is nearly impossible because the intermaxillary correlation is only feasible by manually placing the upper and lower jaw typodonts with risk of misfit. However, the retentive form of the crown is immensely influenced by the convergence angel. In this article, we have proposed a relatively simple method. For this purpose, three lines as 1. long axis of the tooth, 2. overlapping line over the slope of preparation, and 3. connecting the line 1 og 2 (Fig. 4D) will result in a scalene triangle that internal angles can be mathematically calculated and the angle between line 1 and 2 will estimate the convergence angle of the preparation. This function may eventually be coded as a new algorithm to IOS.

Various digital tools facilitate the evaluation of tooth preparation, offering students an objective and immediate assessment of their dexterity skills. One such tool is prepCheck (Dentsply Sirona, Sirona, Bensheim, Germany); however, its implementation demands sufficient equipment and preparation time for the examination (14). The recently introduced E4D Compare software program (Richardson, TX, USA) incorporates a three-dimensional (3D) technique for evaluating prepared teeth. While existing instruments have demonstrated successful outcomes, they rely on high-tech and expensive scanner systems (8). Moreover, these systems necessitate a comparison between teeth prepared by students and those prepared by supervisors as reference, introducing a potential risk of subjective human judgment into the evaluation process. It is noteworthy that these tools are not routinely utilized in daily dental practice by practitioner after graduation. The merit of the method proposed in this article lies in students using intraoral scanners with standard functions supporting both IOS and patient monitoring features. This equips them with competencies applicable in clinical practice. The introduced method ensures excellent alignment in the educational curriculum, enhancing the implementation of pedagogical tools in an effective manner.

While this method proves helpful for instructors in providing constructive feedback to students, it is evident that the current intraoral scanner software has limitations in superimposition, possibly arising from algorithmic constraints and detection limitations (Fig. 4E). The IOS software, specifically in patient monitoring, has demonstrated the ability to detect tooth loss down to 110 µm (13). However, the marked area on the prepared tooth (Fig. 4E) appears to have overgrown after preparation, contrary to expectations. The color mapping (Fig. 4A) indicates tooth reduction below 300 µm, signaling an underprepared area. The accuracy of digital scans is influenced by the operator’s experience (11), indicating that students with limited experience to IOS may use it less effectively. Conversely, younger operators may require less training to enhance their IOS skills. Therefore, the implementation of this function not only facilitates feedback and self-evaluation for students in preclinical teaching but also enhances their proficiency in using IOS in clinical practice. This improvement is crucial for ensuring patient safety and treatment quality, representing an essential integrated element in prosthodontics. Further studies to optimize the model and effect of the method in student’s practical skills and as and preclinical feedback in dental education seem relevant.

## Data Availability

The datasets used and/or analyzed during the current study are available from the corresponding author.
